# Does it matter for the radiologists’ performance whether they read short or long batches in organized mammographic screening?

**DOI:** 10.1007/s00330-021-08010-9

**Published:** 2021-06-10

**Authors:** Heinrich A. Backmann, Marthe Larsen, Anders S. Danielsen, Solveig Hofvind

**Affiliations:** 1 Department of Radiology, Nordland Hospital Trust, Bodø, Norway; 2 Section for Breast Cancer Screening, Cancer Registry of Norway, P.O. Box 5313, 0304 Oslo, Norway; 3 Faculty of Health Sciences, Oslo Metropolitan University, Oslo, Norway

**Keywords:** Mammography, Screening, Radiology, Breast, Sensitivity and specificity

## Abstract

**Objective:**

To analyze the association between radiologists’ performance and image position within a batch in screen reading of mammograms in Norway.

**Method:**

We described true and false positives and true and false negatives by groups of image positions and batch sizes for 2,937,312 screen readings performed from 2012 to 2018. Mixed-effects models were used to obtain adjusted proportions of true and false positive, true and false negative, sensitivity, and specificity for different image positions. We adjusted for time of day and weekday and included the individual variation between the radiologists as random effects. Time spent reading was included in an additional model to explore a possible mediation effect.

**Result:**

True and false positives were negatively associated with image position within the batch, while the rates of true and false negatives were positively associated. In the adjusted analyses, the rate of true positives was 4.0 per 1000 (95% CI: 3.8–4.2) readings for image position 10 and 3.9 (95% CI: 3.7–4.1) for image position 60. The rate of true negatives was 94.4% (95% CI: 94.0–94.8) for image position 10 and 94.8% (95% CI: 94.4–95.2) for image position 60. Per 1000 readings, the rate of false negative was 0.60 (95% CI: 0.53–0.67) for image position 10 and 0.62 (95% CI: 0.55–0.69) for image position 60.

**Conclusion:**

There was a decrease in the radiologists’ sensitivity throughout the batch, and although this effect was small, our results may be clinically relevant at a population level or when multiplying the differences with the number of screen readings for the individual radiologists.

**Key Points:**

• *True and false positive reading scores were negatively associated with image position within a batch.*

• *A decreasing trend of positive scores indicated a beneficial effect of a certain number of screen readings within a batch.*

• *False negative scores increased throughout the batch but the association was not statistically significant.*

**Supplementary Information:**

The online version contains supplementary material available at 10.1007/s00330-021-08010-9.

## Introduction

The aim of mammographic screening is to detect breast cancer in an early stage and reduce breast cancer mortality among asymptomatic women at average risk of breast cancer [1]. A substantial number of women need to be screened to save a small number of women from breast cancer death.

The rate of screen-detected cancer can be influenced by the women’s individual risk of the disease, how the program is organized, and the performance of the radiologists. Menopausal status, use of hormonal treatment, and mammographic density are examples of risk factors [2–5], while screening interval, age range of the target population, and availability to prior mammograms are examples of organizational factors [6]. The radiologists’ experience, time of day, reading volume each day, time spent reading, number of reading sequences (batches), and size of the batches are examples of factors expected to be of influence for the radiologists’ performance [6–11]. However, the evidence concerning the relative importance of these factors is sparse and the studies are often performed in a test or educational setting.

Batch reading without interruptions has been shown to reduce the recall rate without affecting the cancer detection rate [9, 10]. On the other hand, screen reading hundreds of mammograms might cause fatigue, and it is expected that a decrement in vigilance will affect the performance. A recent study demonstrated a decrease in sensitivity and increase in specificity for breast radiologists throughout the day [7]. Based on these findings, we expect a decline in sensitivity throughout a batch of screen readings.

To our knowledge, there is a lack of scientific evidence about the optimal number of screen readings within a batch of mammograms. However, a decrease in sensitivity and an increase in specificity was observed when reading 100 chest x-rays, 60 bone fracture x-rays, and 100 chest CT scans, but the effect was not observed for sequences of 27–50 mammograms [11]. All test sets were enriched and created for the studies. In a cluster randomized controlled trial with a median total batch size of 35 (interquartile range: 16–46) readings, no differences were observed in rates of recall, cancer detection, or disagreement when altering the order of the mammograms for the second reader [12]. Furthermore, the radiologists’ sensitivity did not differ, in contrast to the individual interpretation score, which decreased with increasing image position within the batch.

The European Commission Initiative on Breast Cancer suggests a screening volume between 3500 and 11,000 examinations annually in organized mammography screening programs [6]. Availability to prior mammograms is recommended. The European guidelines from 2006 indicated that the radiologists’ performance deteriorated after 30–40 min [13]. With an average reading time of 20–30 s for each reading, batches should thus not exceed 120 [8, 14, 15]. However, these recommendations are mainly based on expert opinions, and not evidence. Studies aimed at producing this evidence are needed to offer women evidence-based mammographic screening.

In this study, we took advantage of data collected as a part of BreastScreen Norway during the period from 2012 to 2018 and investigated the radiologists’ performance by image position within the batches. We defined performance as true and false positive and true and false negative interpretation scores and sensitivity and specificity of the scores. We hypothesized that performance was associated with image position within the batches.

## Materials and methods

We used data from BreastScreen Norway, a population-based screening program targeting women aged 50–69 [16]. All variables included in the study were collected as a part of a usual screening setting and were extracted retrospectively. The program is administered by the Cancer Registry of Norway and the Cancer Registry Regulation ensures a waiver of the informed consent to perform surveillance, quality assurance, and studies based on the data collected as a part of the program [17]. The data protection officer at the Cancer Registry of Norway (application # 20/12601) approved this study.

BreastScreen Norway offers women two-view mammography of each breast, biennially, hereafter referred to as an examination. Independent double reading is standard and each breast is given an interpretation score from 1 to 5, indicating the suspiciousness of mammographic findings by each radiologist; 1, negative; 2, probably benign; 3, intermediate suspicion of malignancy; 4, probably malignant; 5, high suspicion of malignancy [16]. All examinations with a score of 2 or higher by one or both radiologists are discussed at consensus where the decision to call the woman back for further assessment is made, hereafter referred to as recall. During the 20 first years of screening, the consensus rate was 7%, the recall rate due to positive mammographic findings was 3.2% and the rate of screen-detected cancer was 0.56% [16]. Short-term follow-up is not a standard procedure in BreastScreen Norway.

We considered all readings with a score of 2 or higher independent of laterality, as positive and used the readings, represented by the highest score for each examination, as a unit in the analyses. The standard procedure is to read the mammograms in the same order as they were performed at the screening unit. However, all examinations are sortable and searchable independent of the original order.

**Fig. 1:**
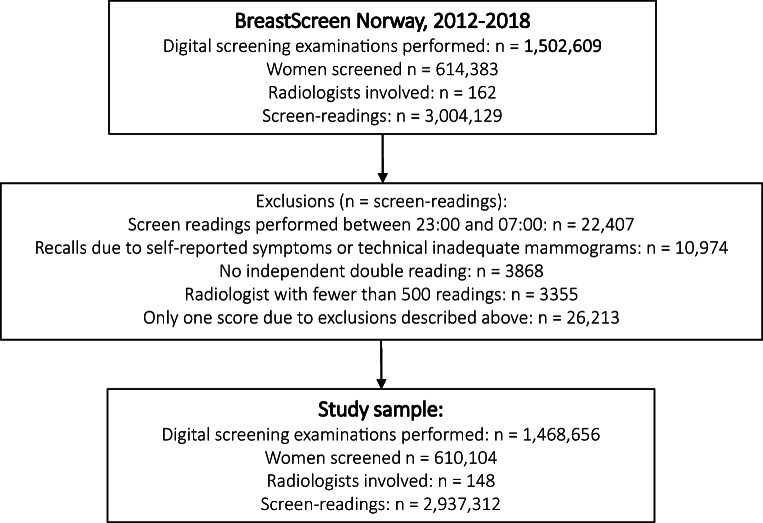
Study population, exclusions, and final study sample

We received a pseudonymized data file with information about 1,502,609 examinations and 3,004,129 screen readings with all digital mammograms performed during the study period, from January 1, 2012, to December 31, 2018. We excluded screen readings performed between 23:00 and 07:00 (*n* = 22,407), readings which resulted in a recall due to self-reported symptoms or technical reasons (*n* = 10,974), readings without double reading (*n* = 3868), and those read by radiologists with less than 500 readings during the study period (*n* = 3355). As a result, some examinations ended up with scores only from one reader. These were excluded (*n* = 26,213). The final study sample included 1,468,656 screening examinations of 610,104 women. All examinations were read by two radiologists, which resulted in 2,937,312 screen readings performed by 148 radiologists (Fig. 1).

### Variables of interest

**Fig. 2:**
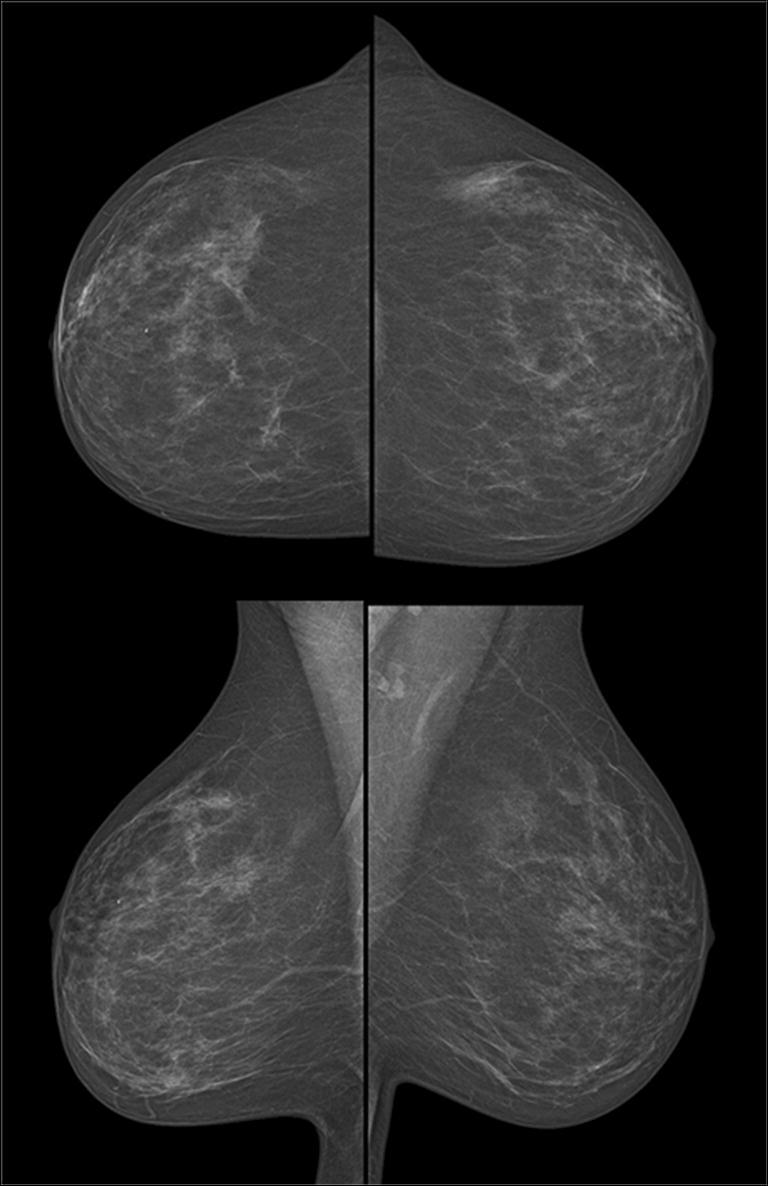
Mammograms (mediolateral oblique and craniocaudal view) of a woman’s right and left breast with an interpretation score of 1 by both radiologists

Each reading was classified as true positive, false positive, true negative, or false negative according to the individual radiologist’s interpretation score and the outcome of the screening examination. Follow-up was related to recall assessment and screen-detected cancers. Interval cancers were not included. An examination was defined per woman and not per breast, which means that the analyses are women-based, not breast-based. A score of 2 or higher by a radiologist to one or both breasts in the examination was defined as a positive score. In this study, we defined true positive as a positive score given to an examination where a cancer (ductal carcinoma in situ or invasive breast cancer) was histologically verified after recall. Mammograms from a woman with an interpretation score of 1 by both radiologists can be seen in Fig. 2. A false-positive reading was defined as a positive score given to an examination, which ended up negative after either consensus or recall, while a true-negative reading was defined as a score of 1 on an examination with a negative outcome. A false-negative reading was defined as a score of 1 on an examination where a cancer was diagnosed after a recall due to a score of 2 or higher by the other radiologist.

The radiologists’ scores were registered with a timestamp, including year, date, and hour, minute, and second, in the database. We defined a batch to be a reading sequence lasting until at least a 15-min break between two following scores while the batch size was defined as the total number of readings within the batch. Image position was defined as the sequential number of the readings within the batch. For descriptive purposes, image position was categorized based on the quartile ranges after defining image positions 1–3 as the first category. The remaining image positions were categorized into 4–16, 17–33, 34–66, and > 66. The batch size was categorized into the following groups based on quartile ranges for batch sizes larger than 1, resulting in five groups: 1, 2–40, 41–72, 73–120, and > 120. Screen reading of only one examination within a batch is hereon referred to as a “single case batch.”

### Statistical methods

Frequencies and proportions were provided for readings, true and false positives, and true and false negatives by each category of image position and total batch size. Proportions of true and false positives and true and false negatives for image positions 1 to 100 were presented with scatter plots, stratified by the first and second reader. Preliminary analyses showed high rates of positive scores for the first image positions versus image positions further in the batch. We assumed this to be due to clinical or mammographic findings disclosed by the radiographers or the first reader and excluded readings with image positions 1–3 in the regression analyses.

Multivariable mixed-effects logistic regression was used to analyze the association between performance measures and image position as a continuous exposure variable. Due to possible confounding, we adjusted for time of day and weekday in the models. Random effects were included for the individual radiologists. Results were presented as adjusted proportions with 95% confidence intervals (CI) for image position 10, 20, 30, 40, 50, 60, 70, 80, 90, 100, 150, 200, and 300. In addition, we explored a possible mediation of the image position effect on performance, by time spent on reading each screening examination (time spent reading). Odds ratios (OR) and 95% CI from models with and without time spent reading are presented in supplementary materials. Stata 16 MP (StataCorp. 2019. Stata Statistical Software: Release 16.1) was used to analyze the data, and the proportion mediated was calculated using the indirect and direct effect from *paramed* in Stata [18].

## Results

Among the 2,937,312 readings, 210,425 (7.2%) were interpreted either first, second, or third within a batch, and 670, 934 (22.8%) had an image position > 66 (Table 1). The true positive rate was 7.2 per 1000 readings for examinations with image positions 1–3 and 3.8 per 1000 readings for image position > 66. The false-positive rate was 6.5% for image positions 1–3, and 3.5% for image position > 66. The rate of true negative was 92.7% for image positions 1–3 and 96.1% for image position > 66, and the rate of false negatives was 0.53 per 1000 for image positions 1–3 and 0.73 per 1000 for image position > 66. This resulted in a sensitivity of 93.2% for image positions 1–3 and 83.7% for image position > 66, and a specificity of 93.4% for image positions 1–3 and 96.5% for image position > 66.

**Table 1:** Frequencies (*n*) and proportions (% or per 1000 readings) of readings, true positive (TP), false positive (FP), true negative (TN), false negative (FN), sensitivity of the radiologists ($\frac{TP}{TP+ FN}\Big)$, and specificity ($\frac{TN}{TN+ FP}$) of the radiologists for each category of image position

Image position	Readings		True positive		False positive		True negative		False negative		$\frac{TP}{TP+ FN}$	$\frac{TN}{TN+ FP}$
	*n*	%	*n*	Per 1000	*n*	%	*n*	%	*n*	Per 1000	%	%
1–3	210,425	7.2	1510	7.2	13,676	6.5	195,128	92.7	111	0.53	93.2	93.4
4–16	715,021	24.3	3084	4.3	34,800	4.9	676,748	94.7	389	0.54	88.8	95.1
17–33	648,840	22.1	2616	4.0	28,506	4.4	617,355	95.2	363	0.56	87.8	95.6
34–66	692,092	23.6	2724	3.9	28,195	4.1	660,735	95.5	438	0.63	86.2	95.9
> 66	670,934	22.8	2530	3.8	23,241	3.5	644,671	96.1	492	0.73	83.7	96.5
Total	2,937,312	100.0	12,464	4.2	128,418	4.4	2,794,637	95.1	1793	0.61	87.4	95.6

**Fig. 3:**
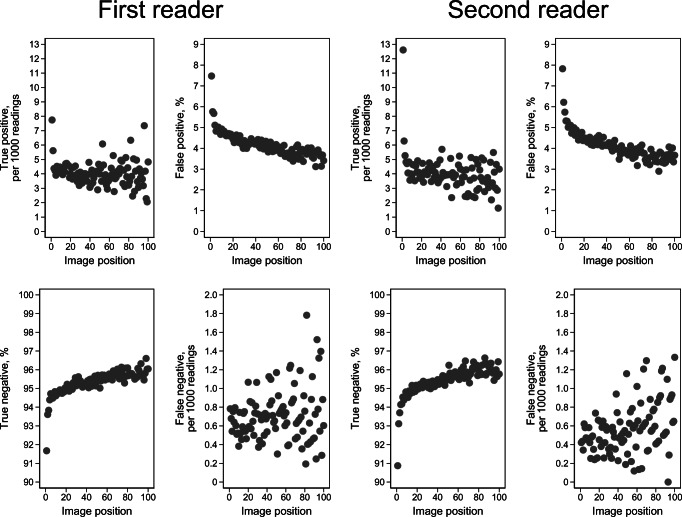
Mean proportion of true positive, false positive, true negative, and false negative for image positions 1 to 100 by the first reader and second reader

The decreasing trend of positive scores and increasing trend of negative scores by image position within the batch were observed for both radiologists. We observed a lower proportion of true positives for reader 1 compared to reader 2 for the first readings, while the proportions did not differ for higher image positions (Fig. 3). Despite the overall trend of higher positive scores for the first readings within a batch, we observed substantial variation between the breast centers in BreastScreen Norway (Supplementary Figure 1).

We identified 74,768 batches, whereof 25,501 included more than 40 readings (Table 2). We found 5577 readings (0.2%) to be read in single case batches. Of those, 266 had a true-positive result, resulting in a true-positive rate of 47.7 per 1000 readings. In the largest batches (> 120 readings), the true-positive rate was 3.6 per 1000 readings. The false-positive rate was 11.7% for single case batches, while it was 3.5% for batches with > 120 readings. The true-negative rate was the lowest in single case batches with 83.5%, while it was 96.1% in batches > 120.

**Table 2:** Frequencies (*n*) and proportions (% or per 1000 readings) of batches, readings, true positive, false positive, true negative, and false negative for each category of total batch size

Batch size	Batches		Readings		True positive		False positive		True negative		False negative	
	*n*	%	*n*	%	*n*	Per 1000	*n*	%	*N*	%	*n*	Per 1000
1	5577	7.5	5577	0.2	266	47.7	655	11.7	4655	83.5	1	0.18
2–40	43,690	58.4	746,303	25.4	3654	4.9	39,929	5.4	702,313	94.1	407	0.55
41–72	13,582	18.2	733,345	25.0	3039	4.1	32,974	4.5	696,912	95.0	420	0.57
73–120	7846	10.5	720,030	24.5	2868	4.0	29,176	4.1	687,522	95.5	464	0.64
> 120	4073	5.5	732,057	24.9	2637	3.6	25,684	3.5	703,235	96.1	501	0.68
Total	74,768	100.0	2,937,312	100.0	12,464	4.2	128,418	4.4	2,794,637	95.1	1,793	0.61

Adjusted analyses showed that image position was associated with true positive, false positive, and true negative (Supplementary Table 1a). For image position 10, the predicted true-positive rate was 4.0 (95% CI: 3.8–4.2) per 1000 readings, for image position 60 the rate was 3.9 (95% CI: 3.7–4.1) per 1000 readings, and for image position 150, the rate was 3.7 (95% CI: 3.4–3.9) per 1000 readings (Table 3). The false-positive rate for image positions 10, 60, and 150 were 5.4% (95% CI: 4.8–5.9), 4.9% (95% CI: 4.4–5.5), and 4.3% (95% CI: 3.8–4.7), respectively. The true-negative rate increased from 94.4% (95% CI: 94.0–94.8) at image position 10 to 94.8% (95% CI: 94.4–95.2) at image position 60 and 95.5% (95% CI: 95.2–95.8) at image position 150. The sensitivity was estimated to be 88.6% (95% CI: 87.6–89.5) for image position 10, 87.7% (95% CI: 86.6–88.7) for image position 60, and 85.9% (95% CI: 84.7–87.0) for image position 150. We found the specificity to be 94.8% (95% CI: 94.4–95.2) for image position 10, 95.2% (95% CI: 94.9–95.6%) for image position 60, and 95.9% (95% CI 95.6–96.2) for image position 150.

**Table 3:** Predicted proportions (% or per 100 readings) of true positive, false positive, true negative, false negative, sensitivity, and specificity of the radiologists for different image positions within a batch of screen readings

Image position	True positive, per 1000	False positive, %	True negative, %	False negative, per 1000	Sensitivity of the radiologists’ scores, %	Specificity for the radiologists’ scores, %
10	4.0 (3.8–4.2)	5.4 (4.8–5.9)	94.4 (94.0–94.8)	0.60 (0.53–0.67)	88.6 (87.6–89.5)	94.8 (94.4–95.2)
20	4.0 (3.7–4.2)	5.3 (4.7–5.8)	94.5 (94.1–94.9)	0.60 (0.53–0.67)	88.4 (87.4–89.4)	94.9 (94.5–95.3)
30	4.0 (3.8–4.2)	5.2 (4.7–5.7)	94.6 (94.2–94.9)	0.61 (0.54–0.68)	88.2 (87.2–89.2)	95.0 (94.6–95.4)
40	4.0 (3.7–4.2)	5.1 (4.6–5.6)	94.6 (94.3 –95.0)	0.61 (0.54–0.68)	88.0 (87.0–89.0)	95.1 (94.7–95.5)
50	3.9 (3.7–4.1)	5.0 (4.5–5.5)	94.7 (94.4–95.1)	0.62 (0.54–0.69)	87.8 (86.8–88.8)	95.2 (94.8–95.5)
60	3.9 (3.7–4.1)	4.9 (4.4–5.4)	94.8 (94.4–95.2)	0.62 (0.55–0.69)	87.7 (86.6–88.7)	95.2 (94.9–95.6)
70	3.9 (3.7–4.1)	4.9 (4.4–5.5)	94.9 (94.5–95.3)	0.62 (0.55–0.70)	87.5 (86.4–88.5)	95.3 (94.9–95.6)
80	3.9 (3.6–4.1)	4.8 (4.3–5.3)	95.0 (94.6–95.3)	0.63 (0.55–0.70)	87.3 (86.2–88.3)	95.4 (95.0–95.7)
90	3.8 (3.6–4.0)	4.7 (4.3–5.2)	95.0 (94.7–95.4)	0.63 (0.56–0.71)	87.1 (86.0–88.1)	95.5 (95.1–95.8)
100	3.8 (3.6–4.0)	4.6 (4.1–5.1)	95.1 (94.8–95.5)	0.64 (0.56–0.72)	86.9 (85.8–88.0)	95.5 (95.2–95.9)
150	3.7 (3.4–3.9)	4.3 (3.8–4.7)	95.5 (95.2–95.8)	0.66 (0.57–0.76)	85.9 (84.7–87.0)	95.9 (95.6–96.2)
200	3.6 (3.3–3.9)	3.9 (3.5–4.3)	95.8 (95.5–96.1)	0.69 (0.57–0.81)	84.8 (83.5–86.0)	96.2 (95.9–96.5)
300	3.4 (3.0–3.8)	3.3 (2.9–3.7)	96.4 (96.1–96.7)	0.74 (0.56–0.92)	82.4 (81.0–83.8)	96.8 (97.1–96.6)

Adjusted models showed that time spent on each reading was associated with true positive, false positive, and true negative (Supplementary Table 1b). The median time spent reading was 35 s (IQR: 21–67) for image position 10, 27 s (IQR: 18–48) for image position 60, and 23 s (IQR: 15–37) for image position 150 (Supplementary Table 2). We estimated that 17% of the total effect of image position on the odds of true positive was mediated through time spent reading. For false positive, the proportion mediated was 8%, and for true negative, the proportion mediated was 9%.

## Discussion

In this study including about 3 million screen readings, we identified image position in the batch to be associated with the radiologists’ performance. The rate of true and false positive was negatively associated while the rate of true negative was positively associated with image position within the batch. The rate of true positive was about two times higher for image positions 1–3 compared to that for image position > 66 (7.2 per 1000 readings vs. 3.8 per 1000 readings). The rate of false negative decreased with image position, but the association was not statistically significant.

The issue of screen reading mammograms in batches has been studied, but the evidence related to an ideal volume within the batches is sparse [8, 12]. Despite finding no overall difference in cancer detection rate when altering the order, the odds of individual recall decreased of the course of examining 40 cases [12]. This is in line with our findings. In addition, we found the modeled reader sensitivity to decrease by image position within the batch, from 88.7% for image position 10 to 87.7% for image position 60, but the clinical implications of the difference are however discussable. A study from the Netherlands defined a batch to be sequential readings until 10 min between two scores and found that image position in a batch was negatively associated with positive scores, but did not report detection rates [8]. Similar to our findings, they also found that reading time was reduced for later image positions within a batch.

Our finding of a substantially higher rate of true positive for the first readings within the batches was unexpected, given the standard workflow and protocol for screen reading in the program. However, if the screen reading is delayed or if there are long waiting times, we are aware that the consecutive reader might be informed about suspicious findings by radiographers or the first reader. This assumption is confirmed in Supplementary Figure 1. Such a practice is not consistent with independent double reading and depletes the remaining mammograms within a batch of cancer cases. On the other hand, such a practice could ensure timely follow-up, diagnostics, and treatment of possible cancer cases in periods of long waiting times. After excluding the first readings in the adjusted proportions, we still found an association between sensitivity and specificity and image position. This indicates that our results are generalizable.

The prevalence of cancer affects the probability of finding true cases [19]. A high prevalence is shown to decrease the rate of false negative compared to a low prevalence setting and is termed “prevalence effect” [20]. The actual prevalence of positive cases among already read images might thus affect the expectation of positive findings for the following readings. This may partly explain the decline in sensitivity for later image positions. The sensitivity is expected to decrease for later image position within the batch because reading a high number of negative cases is expected to affect the probability of identifying positive cases. Due to the low prevalence of cancers in screening, we expect the results from studies based on data from a screening setting, such as ours, to differ somewhat from results from studies based on enriched data sets.

Screen reading of mammograms is an intensive and demanding task including low signal salience, high background stimulus, and high memory workload [21]. Eye-tracking of 13 radiologists reading batches of 40 digital breast tomosynthesis examinations found an increase in blinking after 20 examinations, which indicates fatigue or decreased vigilance [22]. Assuming 70 s per tomosynthesis reading [14, 15], 20 readings correspond to 23 min, which is in line with the meta-analyses by See et al [21]. However, an average reading time of 20–30 s for digital mammography results in about 60 screen readings. The results in our study also might suggest a batch size of about 60–70.

In the future, machine learning and artificial intelligence may help to solve the challenges with numerous negative readings and long batches [23, 24]. However, effective workflow and reading procedures have to be established and implemented when artificial intelligence can be used in a screening setting.

This study of about 3 million screen readings, complete registry data, and readings from 16 breast centers is as far as we are aware the largest study on batch reading in a clinical setting, ever published. Using data from a real-world setting means that the results are transferable to similar screening programs. Furthermore, no maximum batch size or time limitation for screen readings was predefined. These factors represent the strengths of the study. However, the study had limitations. First, a reading could be classified as false negative only if the other radiologist gave a score of 2 or higher. As a result, our false-negative rate is underestimated as some women may had a cancer missed by both radiologists. Second, we had no information about breaks or interruptions shorter than 15 min during the screen reading or the workload distribution among the radiologists. Third, we observed an increased rate of true positive for the first image positions due to variation in the workflow. This depletion of cancers within a batch might affect the radiologists’ performance through an amplified prevalence effect. Furthermore, our study was women-based. Breast-based analyses would be more precise. Lastly, our study did not include information about interval cancers, breast density, mammographic features, or tumor characteristics, which might have influenced our results or interpretation of the results. However, about 25% of the interval cancers were classified as missed in studies where the radiologists retrospectively reviewed prior mammograms [25, 26].

In conclusion, our results might suggest that reading a certain number of screening mammograms within a batch is beneficial when considering the decreasing trend of false-positive scores. However, the decreasing trend of true-positive scores has to be considered. Despite the small differences in the readers’ sensitivity, our results may be of clinical importance at a population level or when multiplying the differences with the number of screen readings for the individual radiologists.

## Supplementary information

ESM 1 Supplementary Figure 1. True positive per 1000 readings for image position 1 to 100 by each of the 16 screening centers in BreastScreen Norway (DOCX 141 kb)

## Abbreviations

CI: Confidence interval
ECIBC: European Commission Initiative on Breast Cancer
IQR: Interquartile range

## Declarations

### Guarantor

The scientific guarantor of this publication is Solveig Hofvind.

### Statistics and biometry

The authors (Larsen, Danielsen) have significant statistical expertise.

### Study subjects or cohorts overlap

The same study population was used in a study published in the *Journal of Medical Screening* with a different aim as in this study. The aim of the other study was the time of day and the radiologists’ performance (https://journals.sagepub.com/doi/abs/10.1177/0969141320953206).

### Methodology

• Retrospective

• Observational

• Multicenter study
