# Supplementary material for: Does it matter for the radiologists’ performance whether they read short or long batches in organized mammographic screening?
Source: Eur Radiol. 2021 Jun 10;31(12):9548–55. doi: 10.1007/s00330-021-08010-9 (PMC8589803; doi:10.1007/s00330-021-08010-9)

Supplementary Table 1. Adjusted odds ratios (OR) and 95% confidence intervals (CI) for positive score, true positive, false positive, true negative and false negative. Numbers marked in bold indicate results that are significant at a significance level of 0.05. Model 1 and model 2 are adjusted for time of day and weekday. The radiologists were included as a random effect

|  | True positive, OR (95% CI) | False positive,  OR (95% CI) | True negative, OR (95% CI) | False negative, OR (95% CI) |
| --- | --- | --- | --- | --- |
| Model 1 |  |  |  |  |
| Image position, per 10 positions | **0.99 (0.99-1.00)** | **0.98 (0.98-0.98)** | **1.02 (1.02-1.02)** | 1.01 (1.00-1.02) |
| Model 2 |  |  |  |  |
| Image position, per 10 positions | **0.99 (0.99-1.00)** | **0.98 (0.98-0.98)** | **1.02 (1.01-1.02)** | 1.01 (1.00-1.02) |
| Time spent reading, per 10 seconds | **1.01 (1.01-1.01)** | **1.01 (1.01-1.01)** | **0.99 (0.99-0.99)** | 1.00 (1.00-1.01) |

Supplementary Table 2. Median time spent reading in seconds with interquartile range (IQR) for different image positions within a batch

| Image position | Median time, seconds (IQR) |
| --- | --- |
| 10 | 35 (21-67) |
| 20 | 33 (20-60) |
| 30 | 31 (19-57) |
| 40 | 30 (18-53) |
| 50 | 29 (18-51) |
| 60 | 27 (18-48) |
| 70 | 27 (17-46) |
| 80 | 26 (17-45) |
| 90 | 25 (17-42) |
| 100 | 25 (16-41) |
| 150 | 23 (15-37) |
| 200 | 21 (13-32) |
| 300 | 19 (10-30) |

Supplementary Figure 1


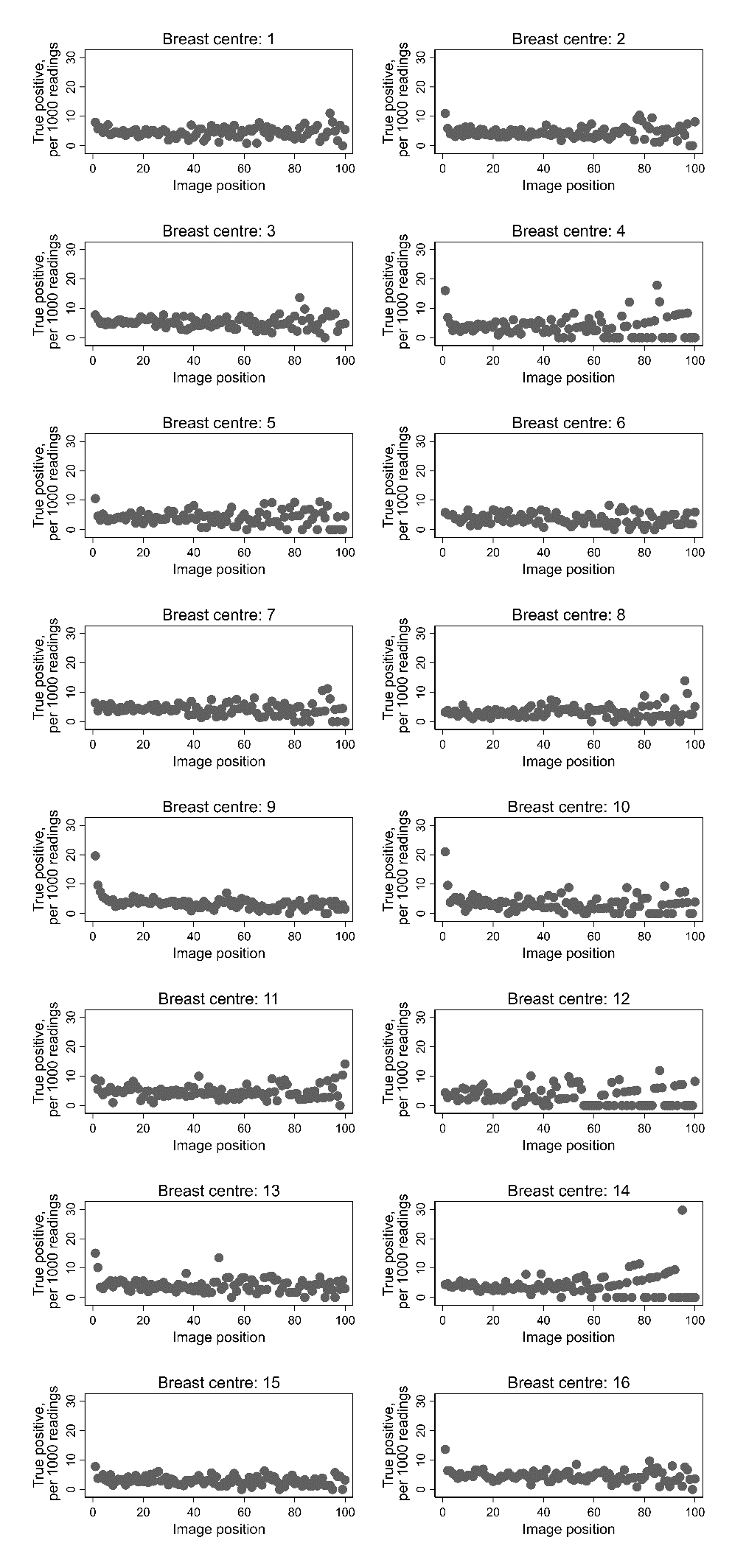

Supplement: Supplementary file 1 — Supplementary Figure 1. True positive per 1000 readings for image position 1 to 100 by each of the 16 screening centers in BreastScreen Norway (DOCX 141 kb) [file 330_2021_8010_MOESM1_ESM.docx]
